# Defining the Ligand Specificity of the Deleted in Colorectal Cancer (DCC) Receptor

**DOI:** 10.1371/journal.pone.0084823

**Published:** 2014-01-06

**Authors:** Patrick C. G. Haddick, Irene Tom, Elizabeth Luis, Gabriel Quiñones, Bernd J. Wranik, Sree R. Ramani, Jean-Philippe Stephan, Marc Tessier-Lavigne, Lino C. Gonzalez

**Affiliations:** 1 Department of Neuroscience, Genentech, South San Francisco, California, United States of America; 2 Department of Protein Chemistry, Genentech, South San Francisco, California, United States of America; School of Biomedical Sciences, The University of Queensland, Australia

## Abstract

The growth and guidance of many axons in the developing nervous system require Netrin-mediated activation of Deleted in Colorectal Cancer (DCC) and other still unknown signaling cues. Commissural axon guidance defects are more severe in *DCC* mutant mice than *Netrin-1* mutant mice, suggesting additional DCC activating signals besides Netrin-1 are involved in proper axon growth. Here we report that interaction screens on extracellular protein microarrays representing over 1,000 proteins uniquely identified Cerebellin 4 (CBLN4), a member of the C1q-tumor necrosis factor (TNF) family, and Netrin-1 as extracellular DCC-binding partners. Immunofluorescence and radio-ligand binding studies demonstrate that Netrin-1 competes with CBLN4 binding at an overlapping site within the membrane-proximal fibronectin domains (FN) 4–6 of DCC and binds with ∼5-fold higher affinity. CBLN4 also binds to the DCC homolog, Neogenin-1 (NEO1), but with a lower affinity compared to DCC. *CBLN4*-null mice did not show a defect in commissural axons of the developing spinal cord but did display a transient increase in the number of wandering axons in the brachial plexus, consistent with a role in axon guidance. Overall, the data solidifies CBLN4 as a bona fide DCC ligand and strengthens its implication in axon guidance.

## Introduction

Extension of axons towards their final target is a major requirement for the proper development of the nervous system. Axons encounter guidance cues along their growth routes, including four major canonical signaling pathways: DCC-Unc5-Netrin-1, Slit-Roundabout (Robo), Semaphorin-Plexin-Neuropilin, and Ephrin-Eph [1]–[3]. However, there is growing appreciation that additional molecules contribute to this process as well [4], [5]. In order to better understand axon growth and guidance, it will be important to determine the repertoire of proteins that bind and interact with these key signaling components.

Here we focus on identifying interacting proteins for DCC. DCC is a 175–190 kDa member of the Ig superfamily consisting of a single transmembrane domain and a large extracellular domain (ECD) containing four C2-like Ig domains and six FNIII domains [6]–[8]. DCC has recently been reported to bind the secreted ligand Draxin in mediating axonal outgrowth inhibition [9]. However, the best-characterized ligand of DCC is Netrin-1, a secreted protein consisting of a globular domain VI, a domain V with three EGF repeats, and a C-terminal domain of unknown function [10]. Although the precise site of Netrin-1 binding to DCC is not known, the fifth FNIII repeat of DCC is required [11], [12].

In *Drosophila*, mutants of the DCC homologue *frazzled* lead to more severe axon midline guidance defects than *netrinAB* mutants and this is partially rescued by *commissureless* independent of netrin and suggestive of an additional frazzled ligand [13]. Similarly, knock-out of either *DCC* or *Netrin-1* in mice results in a failure of commissural axons to cross the spinal cord midline but the severity of the commissural axon defects in the developing spinal cord are greater in *DCC* knock-out mice compared to *Netrin-1* mutant mice [14], [15]. Although there is not a mammalian homologue of *commissureless*, these findings support the suggestion that there are additional ligands regulating DCC activity in vertebrates.

Here we report, using an unbiased large-scale protein-microarray screen, that in addition to Netrin-1, CBLN4 is a highly specific ligand for DCC. The four members of the CBLN family, CBLN1-4, are secreted, glycosylated proteins that contain a C-terminal globular C1q domain characteristic of the C1q-TNF superfamily [16], and they are expressed in the developing and adult brain [17]. CBLN1 and CBLN2 are the best-understood CBLN family members and have been found to stabilize synapses by acting as a trans-synaptic link, binding with Beta-Neurexins of granule neurons and delta 2 glutamate receptors of Purkinje cells in the cerebellum [18]–[21]. However, little is known of the functional role of CBLN4 and there is no obvert behavioral phenotype in a *CBLN4* knock-out mouse [22]. Recently, an independent study also identified DCC as a CBLN4 binding protein using a more limited candidate-based screen [22]. We have further characterized the DCC-CBLN4 interaction and identified a transient wandering-axon phenotype in the brachial plexus in the murine *CBLN4* knock-out.

## Materials and Methods

### Ethics Statement

The Genentech Institutional Animal Care and Use Committee approved all animal studies.

### Cloning and Protein Expression

Expression constructs were generated by standard PCR methods using an in-house clone collection or cDNA purchased from Origene as template and subcloned into pRK5 vector containing the appropriate N-terminal or C-terminal epitope tag. Mutants for DCC domain mapping studies were made using the Quick-Change II XL site-directed mutagenesis kit (Stratagene). All constructs were sequence verified before use. N-terminal flag-tagged (MGGTAARLGAVILFVVIVGLHGVRGKDYKDDDDKLE), full-length and deletion constructs for human DCC are as follows: Flag-DCC full-length (H26-I1442); Flag-DCC Ig1-4 (H26-S425-BamH1-L1098-I1442); Flag-DCC FN1-6 (S426-I1442), Flag-DCC FN4-6 (E722-I1442). Protein expression constructs were generated for soluble (C-terminal domain deleted) [7] human Netrin-1 (G25-K453) with a N-terminal gD-tag (MGGTAARLGAVILFVVIVGLHGVRGKYALADASLKMADPNRFRGKDLPVLLE), human CBLN4 (M1-L201) with a C-terminal Fc tag and the extracellular domain of human DCC (M1-N1097) with a C-terminal Fc tag. Complete amino-acid sequences are provided in Table S1.

Proteins were expressed in Chinese hamster ovary (CHO) cells and purified as described previously [23]. Briefly, the proteins were purified from transient transfection media by batch adsorption onto anti-Flag or anti-gD agarose resin followed by packing of the resin into columns for standard chromatography. Fc-tagged proteins were purified by loading onto a protein A-Sepharose (Amersham Biosciences) column. After washing with Phosphate Buffered Saline (PBS) to baseline, proteins were eluted with 0.1 M acetic acid pH 3 and neutralized immediately with 1.5 M Tris pH 8.6. A secondary gel filtration column was run as a final polishing step. Protein purity was assessed by SDS-PAGE stained with Coomassie Blue and were >90% pure.

### Protein Microarrays

Protein microarrays were constructed on epoxysilane coated glass slides (Schott) as previously described [24]. Two secreted protein libraries were used. Library 1 comprised 1,334 purified samples, representing 686 secreted or extracellular domains of single-transmembrane proteins [25]. The second secreted protein library, Library 2, consisted of 624 samples, representing 562 genes (92 genes were present in both libraries) (Table S2). Details of the screening methods have been previously described [24]. Briefly, multivalent protein-A microbeads (Miltenyi Biotech) were prepared from a 1∶1 mix of DCC-Fc and Cy5-labeled-hIgG and incubated with the microarray slides using an automated aHyb hybridization station (Miltenyi Biotech). Slides were washed with PBS-T (PBS +0.1% Tween20), dried and then scanned for Cy5 fluorescence using a GenePix 4000B scanner (Molecular Devices). Slides were analyzed using GenePix Pro 6.0 software (Molecular Devices). Reported signal intensities were normalized by the average fluorescent signal across all samples on the slide.

### Binding Experiments

COS-7 cells (ATCC) were transfected with cDNAs encoding DCC or, as a control, Nogo receptor (NgR) using PolyFect (Qiagen) according to manufacturer’s recommendations. After incubation at 37°C for 48 h, cells were washed twice in binding buffer (1% heat inactivated normal goat serum (HINGS), 0.05% sodium azide, 2 µg/ml heparin in PBS), and incubated with 20 nM Alkaline Phasphatase (AP), CBLN4-AP, or Nogo 66-AP from conditioned media for 90 min in binding buffer at room temperature. The cells were then washed three times in binding buffer, fixed for 7 min in 4% paraformaldehyde (PFA) in PBS, washed three times in HEPES buffered solution (HBS –20 mM HEPES, pH 7.0, 150 mM NaCl), heat inactivated for 30 min at 65°C, and washed three times in alkaline phosphatase buffer (AP buffer –100 mM Tris, pH 9.5, 100 mM NaCl, 50 mM MgCl_2_). The AP signal was developed with nitro-blue tetrazolium chloride (NBT)/5-bromo-4-chloro-3′-indolyphosphate p-toluidine salt (BCIP) stock solution (Roche) diluted 50 fold in AP buffer in the dark for at least 1 h until the AP dependent formation of blue NBT/BCIP precipitate was visualized.

### Surface Plasmon Resonance

Surface Plasmon Resonance (SPR) experiments were performed on a Biacore 3000 (GE Healthcare) at 25°C using a CM5 sensor chip in 0.01 M HEPES, pH 7.4, 0.15 M NaCl, 0.005% Surfactant P20 (HBS-P) running buffer at a flow rate of 30 µl/min. Full-length N-terminally HA-tagged CBLN1 and CBLN2 were purchased from R&D systems. The extracellular region of Robo3 fused to Fc (Robo3-Fc) and full length CBLN3-Fc and CBLN4 C-terminal His were produced recombinantly (Table S1) and purified using standard affinity chromatography as described above. Cerebellin family members (CBLN1, CBLN2, CBLN3, CBLN4) were injected sequentially at a concentration of 10 µg/ml for 3 min over amine-coupled DCC-Fc or Robo3-Fc on the sensor chip. Dissociation was allowed for 3 min in HBS-P.

### Flow Cytometry

HEK293T cells (ATCC) were transiently transfected with Flag-tagged human DCC or DCC domain mutants using Fugene-6 (Promega) according to manufacturer’s recommendations. Flow cytometric binding experiments were performed in PBS containing 2% FBS at 4°C. Expression of Flag-tagged DCC and domain mutants on the surface of HEK293T cells was confirmed 48 h post-transfection with either an anti-Flag M2 antibody (Sigma) or anti-DCC antibody (Calbiochem) followed by incubation with anti-rabbit IgG or anti-mouse IgG phycoerythrin (PE)- or anti-mouse IgG allophycocyanin (APC)-labeled secondary antibody. Protein binding to Flag-tagged DCC, domain mutants, or non-transfected cells was evaluated by incubating cells for 30 min with recombinant CBLN4-Fc, UNC5C-Fc, gD-Netrin-1 or gD-BTLA at 10 µg/ml. Cells were then washed and binding was detected with either anti-human IgG-PE- or anti-human IgG-APC-labeled secondary antibody or anti-gD and anti-mouse IgG-PE- or anti-mouse IgG-APC-labeled secondary antibody.

### Plate-based Cell Binding and Domain Mapping

DCC domain mapping studies were performed in 384-well imaging and assay plates (Aurora Biotechnologies). For COS-7 transfections, DNA for DCC domain mutant constructs and negative control were dispensed into wells at 60 ng per well. Fugene 6 (Promega) transfection reagent was prepared at a ratio of 3 µl of Fugene to 1 µg of DNA in Opti-MEM (Invitrogen), incubated for 5 min, and mixed with DNA in the 384-well plate and incubated for 15–45 min to allow for complex formation. COS-7 cells were dispensed at a density of 3,000 cells per well into 384-well plates and incubated for 48 h. Transfected cells were washed three times and blocked with PBS containing 1% BSA for 1 h prior to incubation with CBLN4-Fc protein or anti-Flag M2 antibody (Sigma) for 1 h on ice. After washing, cells were fixed in 4% PFA for 30 min at room temperature and protein binding was detected with Alexa Fluor-488 labeled anti-human IgG (Invitrogen) or expression was detected with Alex Fluor-647 labeled anti-mouse IgG (Invitrogen) for 1 h. Fluorescent signal for the entire plate was acquired and analyzed on an Image Express Velos (Molecular Devices). Image analysis was performed using the analysis software for the Image Express Velos. Positively stained cells were counted as those above a background fluorescence intensity threshold and within the particle filter parameters for area and mean signal intensity. Images for publication purposes were collected from the same plate using an INCell Analyzer 2000 (GE Healthcare). The candidate interaction screen was also performed using the above staining protocol.

### Radio-ligand Cell Binding Assay

Radio-ligand binding assays were performed with recombinant CBLN4-Fc and gD-Netrin-1 protein with DCC expressing HEK293T cells in 96-well U-bottom plates in DMEM media containing 2% FBS, 50 mM HEPES, pH 7.2 and 0.1% sodium azide. CBLN4-Fc and gD-Netrin-1 were iodinated with ^125^I-Na using the Iodogen method. Competition reactions were set-up in triplicate with a fixed concentration of iodinated ligand premixed with 3-fold serial dilutions of unlabeled ligand starting at 500 nM followed by addition of DCC cell suspension at a density of 250,000 cells per 200 µl to give a final reaction volume of 250 µl. Reactions were incubated for 2 h at room temperature prior to separation of free from bound iodinated ligand using Millipore multi-screen filter plates (Billerica, MA). Air-dried filters were counted using a Wallac Wizard 1470 gamma counter (PerkinElmer Life Sciences). The binding data were evaluated using NewLigand software (Genentech), which uses the fitting algorithm of Munson and Rodbard to determine the binding affinity [26]. For competition studies, assays were conducted as described above except that the unlabeled ligand was substituted with unlabeled competitor. For blocking studies, control HEK293T cells or HEK293T cells expressing DCC were pre-blocked with unlabeled gD-Netrin-1 (1 µM) or CBLN4-Fc (2 µM) for 1.5 h on ice. Then ^125^I CBLN4-Fc (300 pM) or ^125^I gD-Netrin-1 (52 pM), respectively, was added and allowed to bind for 1 h at 4°C before washing and counting.

### Beta-galactosidase Staining

Whole embryo (E11.5) or 200 micron vibratome sections (E13.5) that were fixed in 4% PFA in PBS were washed three times (20 min each) in washing buffer (2 mM MgCl_2_, 5 mM EGTA, 0.02% Nonidet P-40, and 0.01% sodium deoxycholate in PBS) and the β-galactosidase activity staining was developed in washing buffer with 5 mM potassium ferrocyanide, and 1 mg/ml X-gal in Dimethylformamide (DMF) at 37°C in the dark.

### Immunostaining

TAG-1 immunostaining of spinal cord sections was performed as described [27] using clone 4D7 (Developmental Studies Hybridoma bank) at 1∶200 and Alexa Fluor 594-conjugated anti-mouse IgM (Invitrogen) at 1∶500. Images were acquired on a Zeiss Axioplan 2 microscope. For flat-mount immunostaining, eviscerated E11.5 embryos were fixed in 4% PFA in PBS for 2 h, washed three times in PBS, blocked in 2.5% HINGS and 0.2% Triton X-100 in PBS, and incubated in 1∶2000 neurofilament (NF-M) antibody (Covance) diluted in blocking solution overnight at 4°C. The embryos were washed six times (1 h each) in blocking solution at 4°C and incubated overnight protected from light at 4°C in 1∶200 Alexa Fluor-594-conjugated anti-rabbit IgG (Invitrogen) diluted in blocking solution. The embryos were then washed six times (1 h each) in 0.2% Triton in PBS at 4°C and then cleared in a series of 30%, 50%, and 80% glycerol in PBS washes for at least 6 h each. The embryo was then flattened between two glass coverslips and imaged using a Zeiss LSM510 confocal microscope.

### Mouse Strains

*Netrin-1* [15] and *CBLN4* [28] knock-out mice were maintained on a CD-1 background and genotyped as described.

## Results

### Identifying DCC Binding Partners

**Figure 1:**
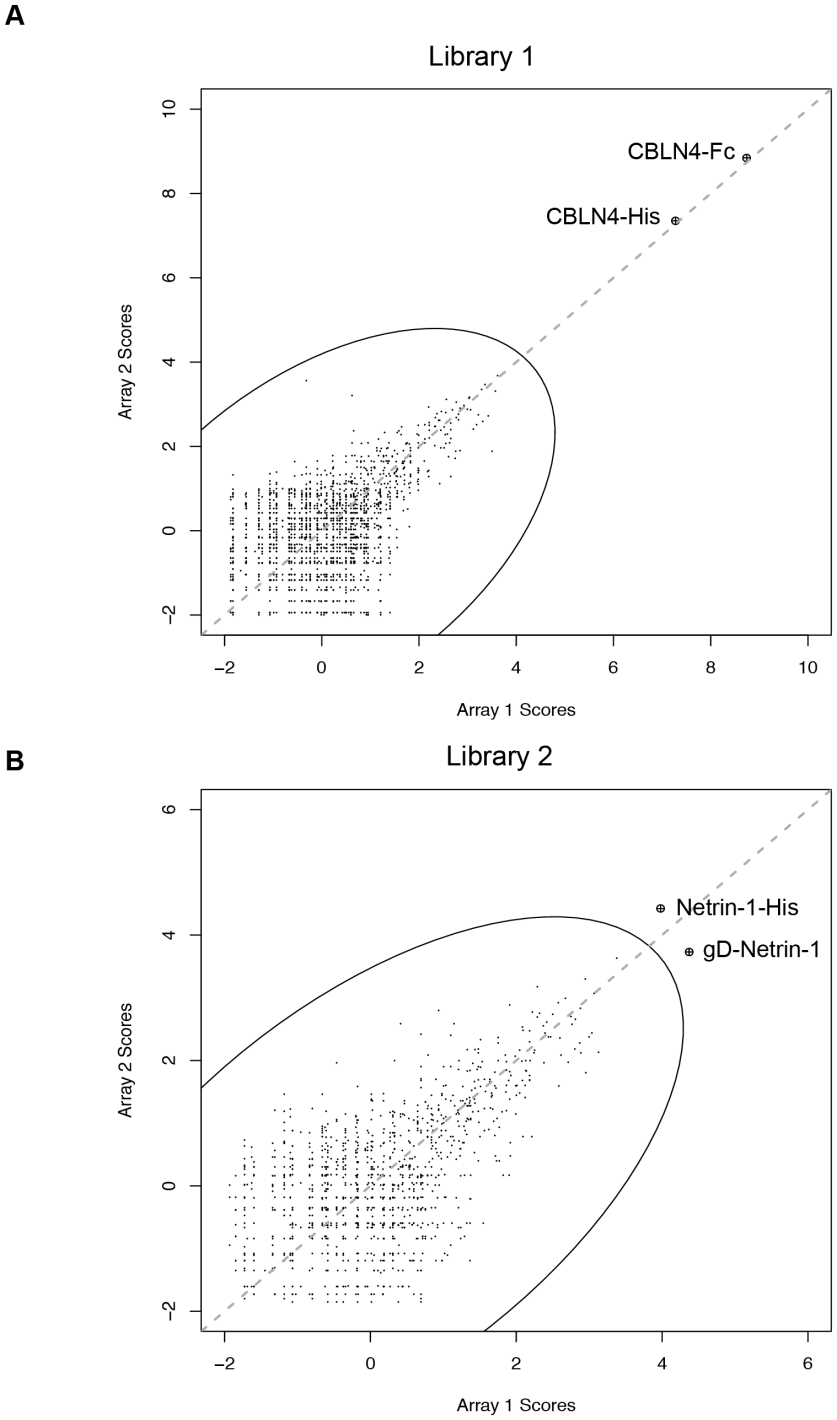
Protein microarrays identify Netrin-1 and CBLN4 as specific DCC binders. The intersection plots show data from two replicate screens (Array 1 and Array 2) of DCC-Fc against two extracellular protein-microarray libraries, representing 686 genes in Library 1 (A) and 562 genes in Library 2 (B). Each point represents a protein sample and the oval represents the cutoff for selected hits. CBLN4-Fc, CBLN4-His, Netrin-1-His and gD-Netrin-1 proteins were the top scoring hits in each library.

To identify candidate binding partners for DCC, an extracellular protein microarray [24], [25] was screened with a multivalent protein-A microbead complex generated from a 1∶1 mix of fluorescently labeled (Cy5) human IgG and the extracellular domain of DCC (amino acids 1–1097) fused to the Fc portion of human IgG. Of the 1,334 protein samples, representing 686 genes, on the microarray, only spots corresponding to CBLN4-Fc and CBLN4-His showed strong fluorescent signals (Figure 1A). A second protein library consisting of 624 protein samples (Table S2), representing 562 genes identified gD-Netrin-1 and Netrin-1-His as the top hits, albeit with lower signal-to-noise ratios compared to CBLN4 (Figure 1B). Netrin-1 was only present in the second library, whereas CBLN4 was only present in the first library. Related family members, CBLN2 and CBLN3, were represented but showed no binding to DCC. Furthermore, three lots of Draxin (C1orf187) were included within the first library but were not identified as hits. In follow-up studies, we tested DCC-Fc for binding to these three Draxin protein samples along with a commercial sample (R&D Systems) by SPR. We were unable to detect direct binding of the recombinant ECD of Draxin to DCC-Fc with any of these samples (data not shown).

**Figure 2:**
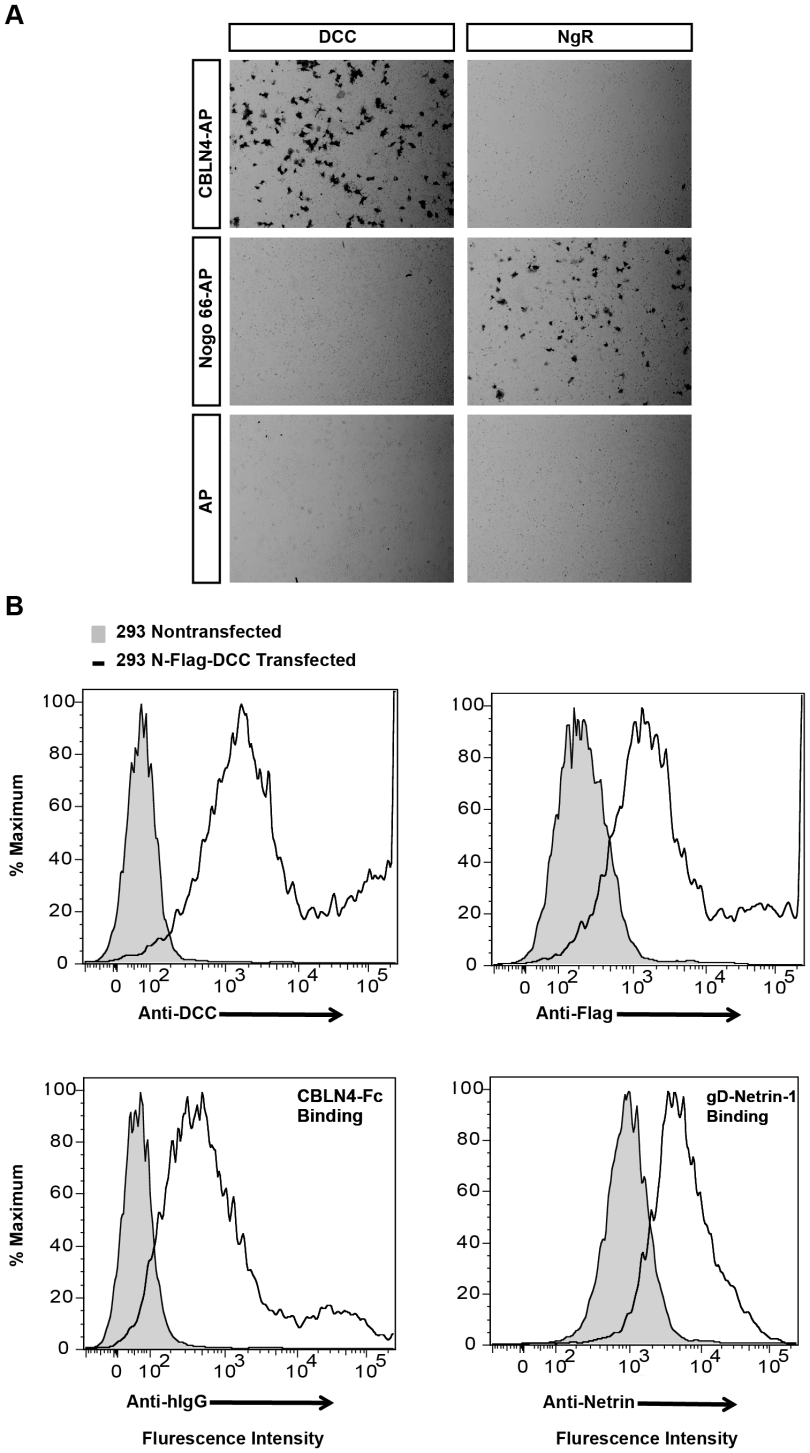
CBLN4 binds specifically with DCC on cells. A) Representative images of CBLN4-AP protein binding to COS-7 cells transfected with DCC, but not NgR expression constructs (top panels). Nogo 66-AP protein bound to NgR but not to DCC transfected cells (middle panels). AP did not interact with either DCC or NgR (lower panels). B) Flow cytometric analysis confirmed cell-surface expression of transiently transfected Flag-tagged DCC on HEK239T cells (top panels). The CBLN4-Fc and gD-Netrin-1 proteins bound to DCC expressing cells as demonstrated by the shift in fluorescent intensity compared to untransfected cells (bottom panels).

To confirm the DCC-CBLN4 interaction on the cell surface, an alkaline phosphatase (AP) binding assay was performed. The results show that CBLN4-AP binds to COS-7 cells transiently transfected with DCC but not to cells transiently transfected with NgR that bind to Nogo66-AP (Figure 2A). In addition, flow cytometry confirmed binding of CBLN4-Fc to HEK293T cells expressing DCC relative to control HEK293T cells (Figures 2B, S1).

**Figure 3:**
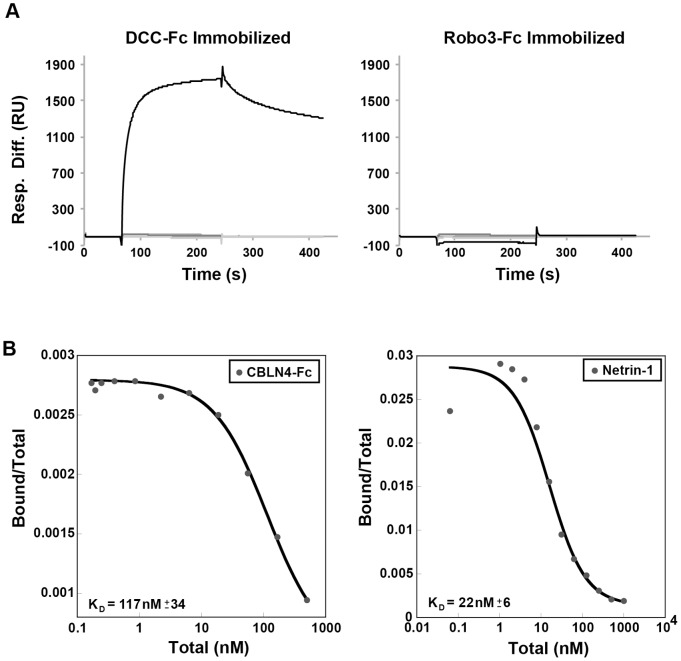
CBLN family binding and cell-based DCC interaction affinity measurements. A) Sensograms are shown from SPR analysis of immobilized DCC-Fc (left; 15,082 response units) and negative control Robo3-Fc (right; 9,833 response units) with CBLN1, CBLN2, CBLN3 and CBLN4 injected as analytes at 10 µg/ml. Robust binding was detected to CBLN4 (black trace), while no binding signal was detected for the other Cerebellin family members (gray traces). B) A radio-lignad assay was used to determine the affinity of CBLN4-Fc (left) and gD-Netrin-1 (right) to DCC transiently transfected into HEK293T cells. ^125^I-Labeled ligand was allowed to bind transfected cells in the presence of increasing amounts of unlabeled ligand. Calculated *K_D_* values are indicated on the graphs and are representative of 3 independent experiments.

To determine if DCC binds exclusively to CBLN4, we evaluated the ability of DCC to interact with other CBLN family members by SPR. The sensograms for the CBLN family members injected over the immobilized DCC or negative control, Robo3, are shown in Figure 3A. A sharp increase in response units (RU) followed by a slow dissociation was observed for CBLN4-His binding to DCC but not to Robo3. By contrast, CBLN1, CBLN2, and CBLN3 did not interact with either DCC or Robo3, demonstrating selective interaction of DCC with CBLN4. The relative binding affinities of CBLN4 and Netrin-1 to transiently transfected HEK293T cells expressing DCC were determined from a radio-ligand cell-binding assay using ^125^I-labeled CLBN4-Fc and gD-Netrin-1. The equilibrium dissociation constant, *K_D_*, for CBLN4 was calculated to be 117±34 nM compared to a dissociation constant of 22±6 nM for Netrin-1 (Figure 3B).

### Identifying CBLN4 Binding Partners

**Figure 4:**
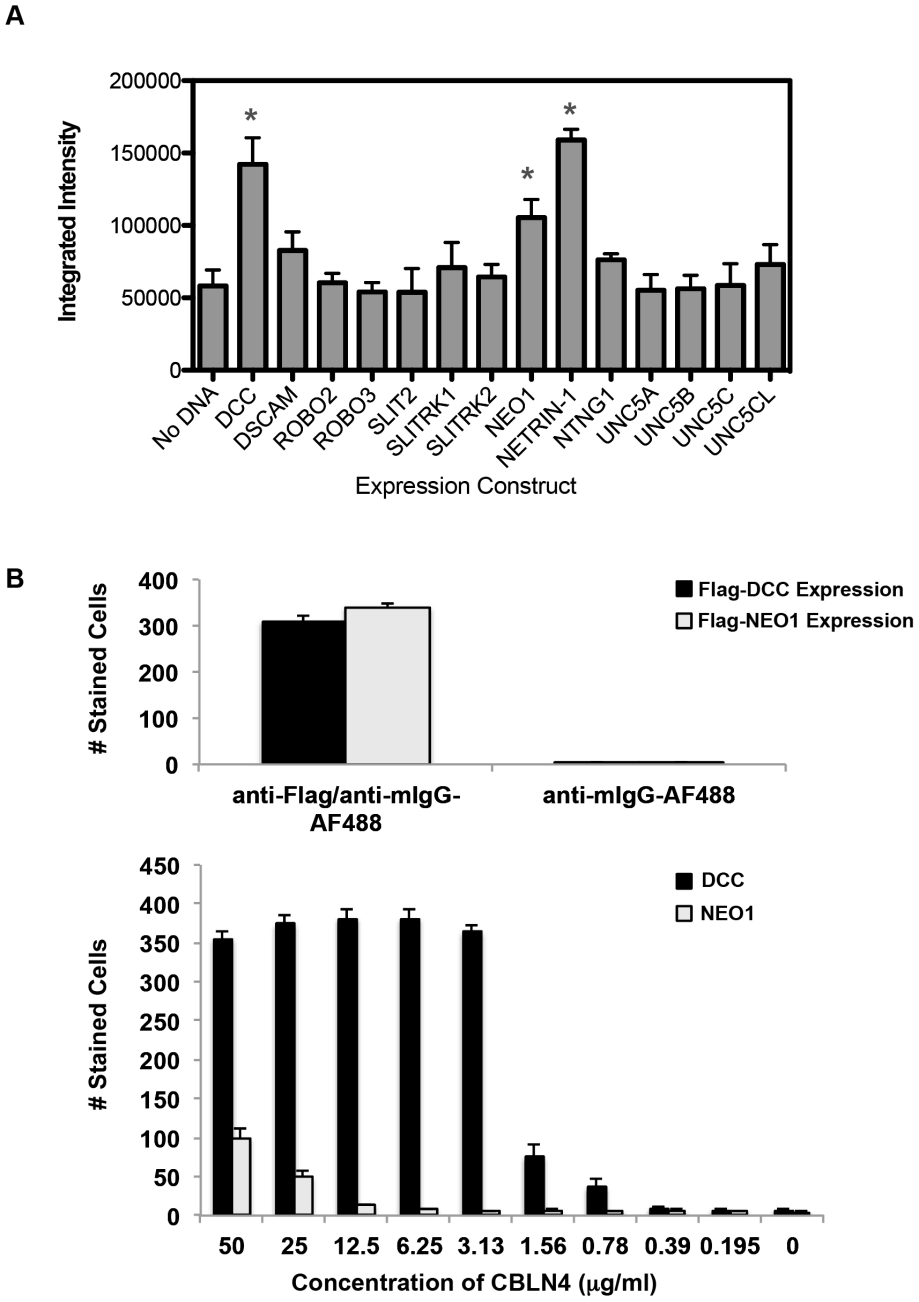
Characterizing CBLN4 binding partners. A) quantification of CBLN4-Fc binding to COS-7 cells transfected with the indicated axon guidance proteins. Data represent three independent replicate plates, each containing triplicate wells and error bars represent standard error of the mean. B) Flag-DCC and Flag-NEO1 were transiently expressed at the surface of COS-7 cell as detected by anti-Flag staining (top panel). The lower panel shows a titration series of CBLN4-Fc binding to DCC or NEO1. Triplicate independent experiments were performed and error bars represent the standard error of the mean.

To determine if there were other CBLN4 receptors besides DCC, a protein-microarray screen was performed in reverse where CBLN4-Fc was used as bait, however, no clear hits were found (data not shown). Curiously, DCC itself was not identified as a hit. Prior studies indicate that proteins are largely active on the microarray substrate [24], although there is the possibility that DCC is sensitive to this type of immobilization. Next, fourteen neuronal proteins, known to coordinate neuron axonal path finding similar to DCC function, were screened using a cell-binding assay. Binding of CBLN4-Fc was evaluated against COS-7 cells transfected with clones corresponding to untagged full-length candidate genes. It should be noted that a negative result does not necessarily rule out the candidate interaction since protein expressions were unconfirmed. Quantification of the fluorescent intensity for CBLN4-Fc staining to these transfected clones identified DCC and two other potential ligands, Netrin-1 and NEO1 (Figure 4A). The signal observed for Netrin-1 binding, however, remained constant regardless of changes in CBLN4 concentration and also with secondary detection antibody alone (data not shown), indicative of a false positive in the screen. NEO1, interestingly, is a DCC paralogue and also functions as a Netrin -1 receptor [29]. Both DCC and NEO1 have similar domain structures and share 54% identity over the ECD region. To qualitatively assess the binding strengths to Netrin-1 and NEO1, DCC-expressing COS-7 cells were individually evaluated with a titration series of decreasing CBLN4-Fc. The interaction between CBLN4 and NEO1 could be detected at CBLN4-Fc concentrations of 25 µg/ml (280 nM) or higher. In contrast, CBLN4-Fc binding to DCC-expressing cells showed robust signals with concentrations as low as 0.78 µg/ml (8.7 nM) (Figure 4B). These data suggest that binding of NEO1 to CBLN4 represents a much lower affinity interaction compared to DCC binding CBLN4. To further characterize the CBLN4-NEO1 interaction, NEO1 transient expressing HEK293T cells were analyzed for binding to CBLN4-Fc protein by flow cytometry and radio-ligand binding assay using ^125^I-labeled CBLN4. However, CBLN4 did not interact with NEO1 expressing cells with either of these approaches, likely because of a very low affinity (data not shown).

### CBLN4 Binds to the FN4-6 Region of DCC

**Figure 5:**
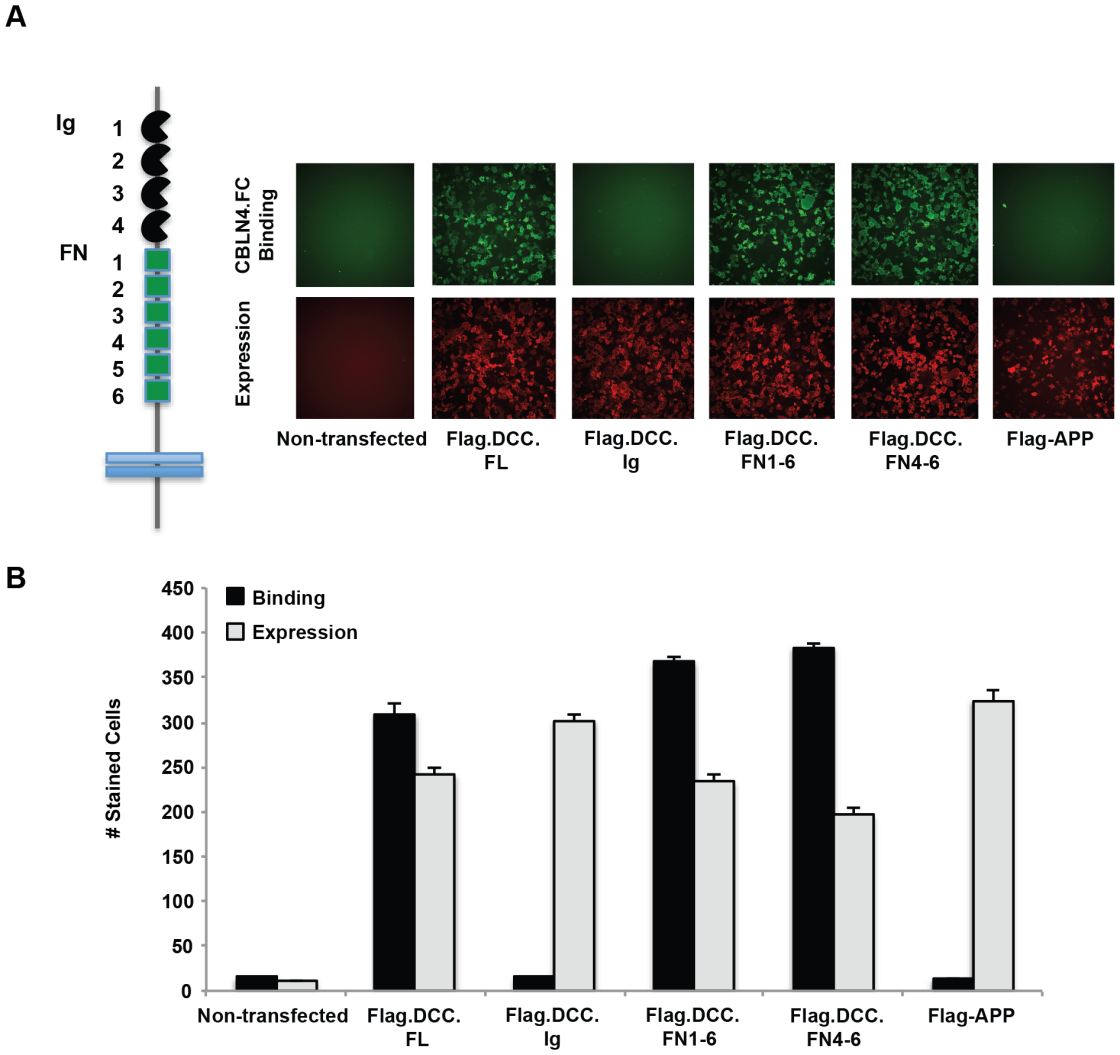
CBLN4 binds to the FN4-6 region of DCC. A) a schematic representation of DCC is shown with the Ig domains in black and the FN domains in green. Representative fluorescent images are shown for CBLN4-Fc binding to COS-7 cells expressing full-length DCC, DCC Ig1-4, DCC FN1-6, DCC FN4-6, and negative control APP. Detection of CBLN4-Fc binding was through anti-human Alexa Fluor-488 (green). Detection of cell surface expression of all flag-tagged DCC deletion mutants was confirmed with an Alex Fluor-647 labeled anti-mouse IgG (red). B) quantification of CBLN4-Fc binding to the DCC domain deletion constructs. Triplicate independent experiments were performed and error bars represent the standard error of the mean.

DCC is a single transmembrane protein containing four Ig repeats and six FNIII repeats in the ECD. To assess which domains in DCC are necessary for CBLN4 binding, deletion constructs of DCC were generated and transfected into COS-7 cells for binding analysis. Four DCC constructs were tested, constituting either the entire ECD (DCC.FL), the Ig1-4 repeats (DCC.Ig), the FN1-6 repeats (DCC.FN1-6), or only the C-terminal FN4-6 repeats (DCC.FN4-6). The Ig1-4 repeats (DCC.Ig) showed no binding to CBLN4-Fc, suggesting that primary binding is dependent on the FN domains (Figures 5A–B). Consistent with this interpretation, expression of only the FN repeats (DCC.FN1-6) or only the last three FN domains (DCC.FN4-6) retained strong CBLN4-Fc binding. These results demonstrate that DCC-FN4-6 is sufficient to mediate binding to CBLN4.

### Netrin-1 Competes with CBLN4 Binding

**Figure 6:**
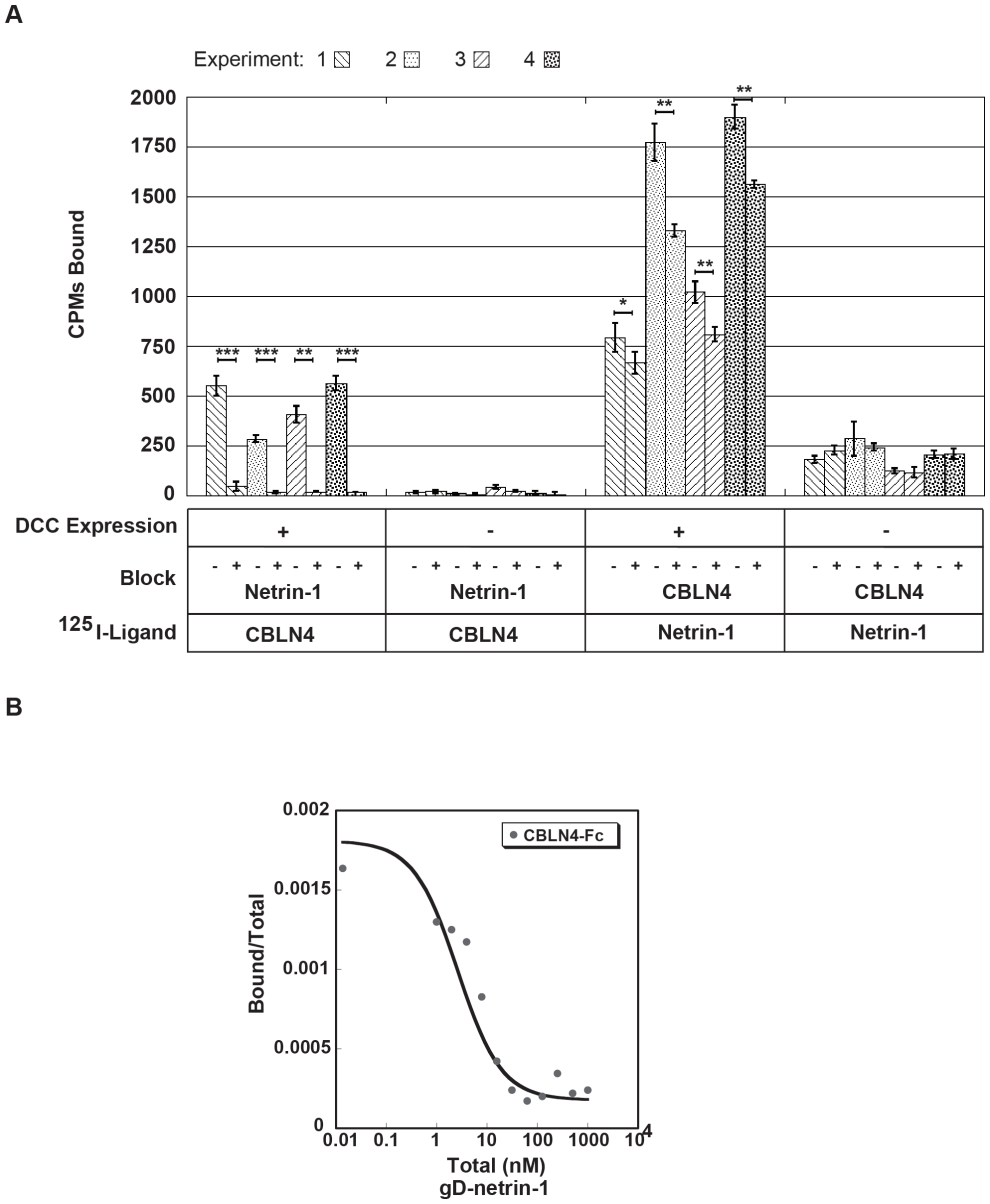
Netrin-1 competes CBLN4 binding to DCC. A) quantification of the amount of ^125^I-radiolabeled CBLN4-Fc or gD-Netrin-1 bound to DCC expressing HEK293T cells, or untransfected control cells, preincubated with unlabeled gD-Netrin-1 or CBLN4-Fc, respectively. Data from four independent experiments are shown. Each bar represents 3 technical replicates expressed as means ± SD. One-tailed Student’s *t* test were applied: *, *P*<0.05; **, *P*<0.01; ***, *P*<0.001. B) displacement plot, representative from triplicate independent experiments, is shown for gD-Netrin-1 competing with binding of ^125^I-radiolabeled CBLN4-Fc to DCC expressing HEK293T cells.

Previously published reports implicate the FN4-6 region of DCC in Netrin-1 binding [11], [12]. Our observation that CBLN4 binding mapped to the same region on DCC led us to examine if Netrin-1 and CBLN4 compete for DCC binding or if all three proteins could form a ternary complex. Using co-immunoprecipitation studies, it has been recently reported that CBLN4 binding to DCC can be competed by Netrin-1 [22]. To verify these results, we utilized a sensitive radio-ligand based approach. The data show that ^125^I-labeled CBLN4-Fc (300 pM) bound specifically to DCC expressing HEK293T cells and that binding was blocked by pre-incubation of the cells with Netrin-1 (1 µM) compared to no treatment control (Figure 6A). Incomplete blocking of ^125^I-labeled Netrin-1 (52 pM) with CBLN4-Fc (2 µM) was observed in the reverse experiment and most likely can be attributed to the ∼5-fold difference in affinity of CBLN4 and Netrin-1 for DCC. A titration with increasing concentrations of Netrin-1 showed clear displacement of a fixed concentration of ^125^I CBLN4-Fc to DCC expressing HEK293T cells (Figure 6B). Our data demonstrate that CBLN4 and Netrin-1 binding sites map to the same FN region in DCC and sterically overlap.

### Characterization of CBLN4 Function during Embryonic Development

**Figure 7:**
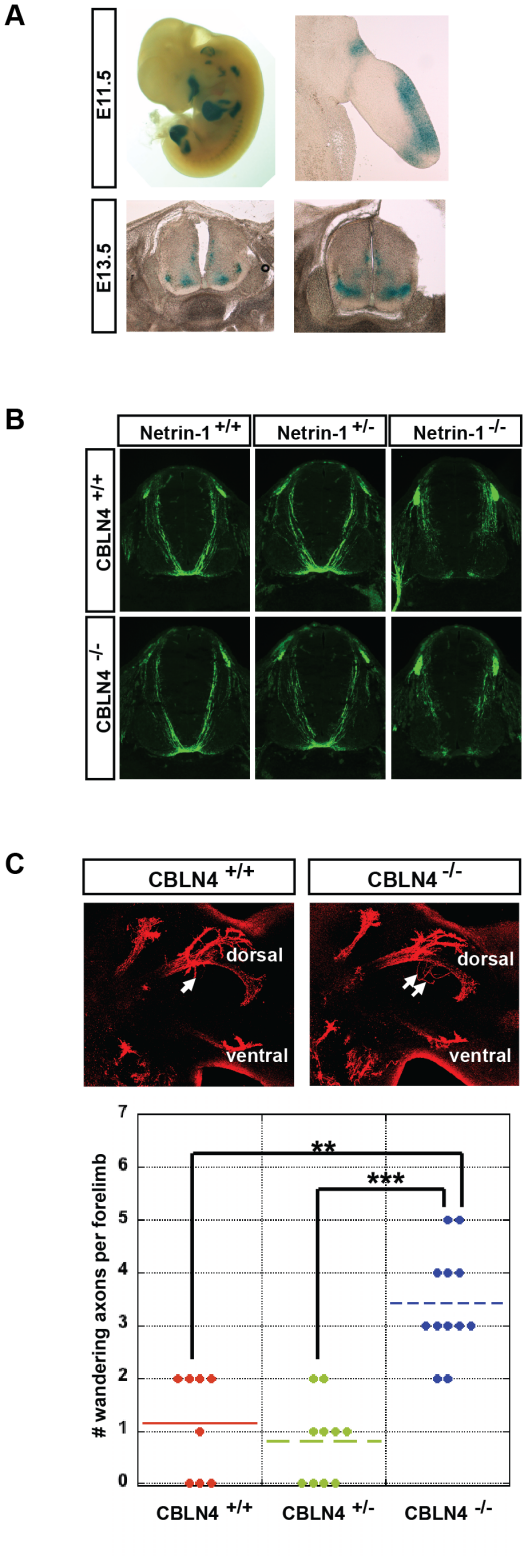
Distinct expression pattern of CBLN4 during embryonic development and subtle effects on axon pathfinding in *CBLN4−/−* mice. A) Lac-Z reporter expression of CBLN4 in E11.5 *CBLN4^+/−^* mice (top panels) showing distinctive expression in limbs including dorsal limb expression. Lac-Z reporter staining of CBLN4 in E13.5 *CBLN4^+/−^* mice (lower panels) showing expression in the spinal cord motor column. B) commissural axons of E11.5 spinal cord visualized with TAG-1 immunostaining in combinations of *CBLN4* and *Netrin-1* genotypes. C) Representative images and quantification of wandering axons (white arrows) in the brachial plexus of E11.5 *CBLN4^−/−^* mice. Tukey contrasts; **, *P*<0.01; ***, *P*<0.001; n is left and right forelimb nested in mouse; n = 4 *CBLN4^+/+^*; n = 5 *CBLN4^+/−^*; n = 6 *CBLN4^−/−^*.

It has previously been shown that DCC and Netrin-1 are necessary for the proper guidance and growth of axons during embryonic development [7], [15]. In order to examine the contribution CBLN4 may have in DCC-Netrin-1 signaling, the neuronal projections of *CBLN4* knock-out mice were studied. Generation of the *CBLN4* knock-out mouse included a knock in of a LacZ expression cassette that allows β-galactosidase staining of *CBLN4^+/−^* mice to visualize the localization of CBLN4 expression. The β-galactosidase expression pattern in *CBLN4^+/−^* mice is distinct, including expression in the dorsal limb buds at E11.5 and the motor column of the spinal cord at E13.5 (Figure 7A). One of the major roles for DCC and Netrin-1 is ensuring the proper midline crossing of commissural axons in the developing spinal cord. To examine if CBLN4 plays a role in midline guidance, pre-crossing commissural axons were visualized by TAG-1 immunostaining in transverse sections of E11.5 mice (Figure 7B *)*. Whereas the commissural axons can be seen reaching and crossing the floor plate in wild type mice, very few, if any, commissural axons reach and cross the floor plate in *Netrin-1^−/−^* mice. TAG-1 immunostaining of commissural axons of *CBLN4^−/−^* mice show that they reach and cross the axons similar to wild type and there is no noticeable difference in immunostaining between *Netrin-1^−/−^*;*CBLN4^+/+^* and *Netrin-1^−/−^*;*CBLN4^−/−^* mice or *Netrin-1^+/−^*; *CBLN4^+/+^* and *Netrin-1^+/−^*; *CBLN4^−/−^* mice.

The expression of CBLN4 in developing limb buds led us to evaluate the trajectory of motor neuron axons at this stage. These axons express DCC [7] and navigate from the motor column of the spinal cord to their final targets innervating the muscles in the limb [30] and are known to be responsive to Netrin-1 [31], [32]. Motor axon projections in *CBLN4^−/−^* were visualized by confocal images of flatmounted limbs immunostained for Neurofilament. A significant increase in the number of wandering axons was detected in the dorsally projecting bundle in the E11.5 brachial plexus of *CBLN4^−/−^* mice compared to *CBLN4^+/+^* and *CBLN4^+/−^* embryos (Figure 7C). This effect, however, was transient as no increase in the number of wandering axons in the E13.5 brachial plexus was observed (data not shown).

## Discussion

Axon guidance receptors generally display complex temporal-spatial expression patterns and often utilize different ligands [33], [34]. For example, UNC5B, originally characterized as a Netrin-1 receptor, was recently found to bind to Robo4 on endothelial cells and can also bind to the FLRT receptor family, mediating repulsive axon guidance [35]–[37]. To help our understanding of axon guidance, the full complement of extracellular signaling molecules must be uncovered. The application of technologies for screening extracellular protein-protein interactions is an emerging approach for identifying unknown axon-guidance molecules [38]. In one example, a screening methodology called AVEXIS was used to interrogate the interactions among 150 zebrafish receptors from the leucine-rich-repeat and/or Ig superfamily expressed in the nervous system. The results outlined a network of 34 receptor-ligand pairs, including novel interacting partners for the well-characterized axon guidance receptors Robo2 and Robo3 [36]. These candidate interactions serve as a starting point for further studies aimed at understanding their *in vivo* functional roles.

Here we have utilized an extracellular protein microarray that allows efficient screening of nearly one-third of the known human secreted or cell-surface expressed, single-transmembrane-containing genes [24], [38]. Prior work has shown that the microarray approach is able to robustly identify the majority of expected interactions within the Ig receptor family, including those having low binding affinities [24], [39]. Using this platform, we set out to evaluate binding partners for DCC, a well-study receptor in axon guidance. We were motivated by the observation that commissural axon crossing of the midline does not phenocopy in the *DCC* and *Netrin-1* knock-out mice. *DCC* mutants have a more severe defect, suggesting that DCC may harbor yet unidentified ligands. Interaction of DCC against 1,156 extracellular proteins was tested and the top hits corresponded to Netrin-1 and CBLN4. This large-scale screen serves to further reinforce the high degree of specificity and biochemically validates the DCC-CBLN4 interaction [22].

Although CBLN4 and Netrin-1 bind to DCC in the same region of the extracellular domain, the relative affinities differ by approximately 5-fold. In the developing mouse embryo, there are distinct regions of CBLN4 and Netrin-1 expression as well as overlapping regions such as the limb bud [40]. It is expected the higher affinity of Netrin-1 would lead to Netrin-1 dependent activity dominating CBLN4 specific activity, but there could still be instances of much higher CBLN4 concentrations relative to Netrin-1 that would affect Netrin-1 signaling. For these reasons, it will be important to characterize the relative expression profiles of Netrin-1 and CBLN4 as well as their functional effects on DCC signaling. Moreover, there is evidence CBLN4 can heterodimerize with CBLN1 [22] and this may indirectly affect the binding of CBLN4 and Netrin-1 to DCC.

NEO1 is structurally and functionally similar to DCC [10]. Despite the much lower apparent affinity to NEO1, our data suggests that a functional interaction with CBLN4 cannot be ruled out. Given the predicted oligomeric/multimeric structure of CBLN4 [16] *in vivo*, the avidity or high local concentrations might compensate for the observed low-affinity binding.

One of the most studied systems to interrogate DCC’s function is the midline crossing of commissural axons in the developing spinal cord where both DCC and Netrin-1 are required for proper crossing [14], [15]. However, we did not observe defects in commissural axons in the absence of CBLN4. The earliest we detected CBLN4 gene expression in the spinal cord was E13.5, an age when most pioneering commissural axons have already crossed the midline [31]. There were no other DCC-binding ligand candidates from the protein microarray that may account for the more severe commissural axon defects in *DCC^−/−^* mice compared to *Netrin-1^−/−^* mice. We cannot exclude that there was a false negative or that the ligand was not present in the protein microarray library. Alternatively, the increased severity of *DCC^−/−^* commissural axon defects compared to *Netrin-1^−/−^* could originate from DCC dependent loss of signaling that is not mediated by ligand binding to extracellular DCC.

CBLN4 expression is most robust in the motor column of the developing spinal cord and limb buds. Given this expression, we examined the possibility of CBLN4 being involved in regulating the innervation of motor axons to their targets in the developing muscles of the limb buds. Indeed, we did see a modest increase in wandering axons in the dorsal bundle of the brachial plexus that consist of motor axons that project to dorsal limb muscles. This increase in wandering axons was transient, not detected in embryos older than E11.5, and there are no reports of motor defects in adult *CBLN4^−/−^* mice to suggest severe motor axon innervation defects [22]. CBLN4 also has a distinctive expression pattern in the developing limb. *Lmx1b^−/−^* mice have a loss of dorsal limb structures and a microarray study found CBLN4 transcripts are lower in *Lmx1b^−/−^* dorsal limb buds compared to wild type dorsal limb buds [41]. The wandering axons in *CBLN4^−/−^* embryos strayed from axons that ultimately innervate the dorsal limb and it may be that CBLN4 is involved in dorsal limb specification that leads to an increase in wandering dorsal axons in the absence of CBLN4. DCC and Netrin-1 are also expressed in the developing limb bud [40] and it will be interesting to explore if they also contribute to the wandering axons seen in *CBLN4^−/−^* mice.

## Supporting Information

Figure S1 **Flow Cytometric analysis of CBLN4 and Netrin-1 binding to DCC expressed on HEK293T cells.** Column 1 demonstrates cell-surface expression for each construct. Columns 2 and 3 show binding of CBLN4 and negative control protein UNC5C to various DCC constructs. UNC5C shows low-level binding to untransfected HEK293T cells and shows no increased binding to DCC. Columns 4 and 5 show binding of Netrin-1 and control protein BTLA to various DCC constructs. Netrin-1 and CBLN4 show similar DCC-binding profiles.
(TIF)

Table S1 **Domain boundaries and amino acid sequences for protein reagents used in this study.**
(XLS)

Table S2 **Composition of the libraries used for the protein microarray screens.**
(XLS)
