# Supplementary figures and images for: Defining the Ligand Specificity of the Deleted in Colorectal Cancer (DCC) Receptor
Source: PLoS One. 2014 Jan 6;9(1):e84823. doi: 10.1371/journal.pone.0084823 (PMC3882260; doi:10.1371/journal.pone.0084823)

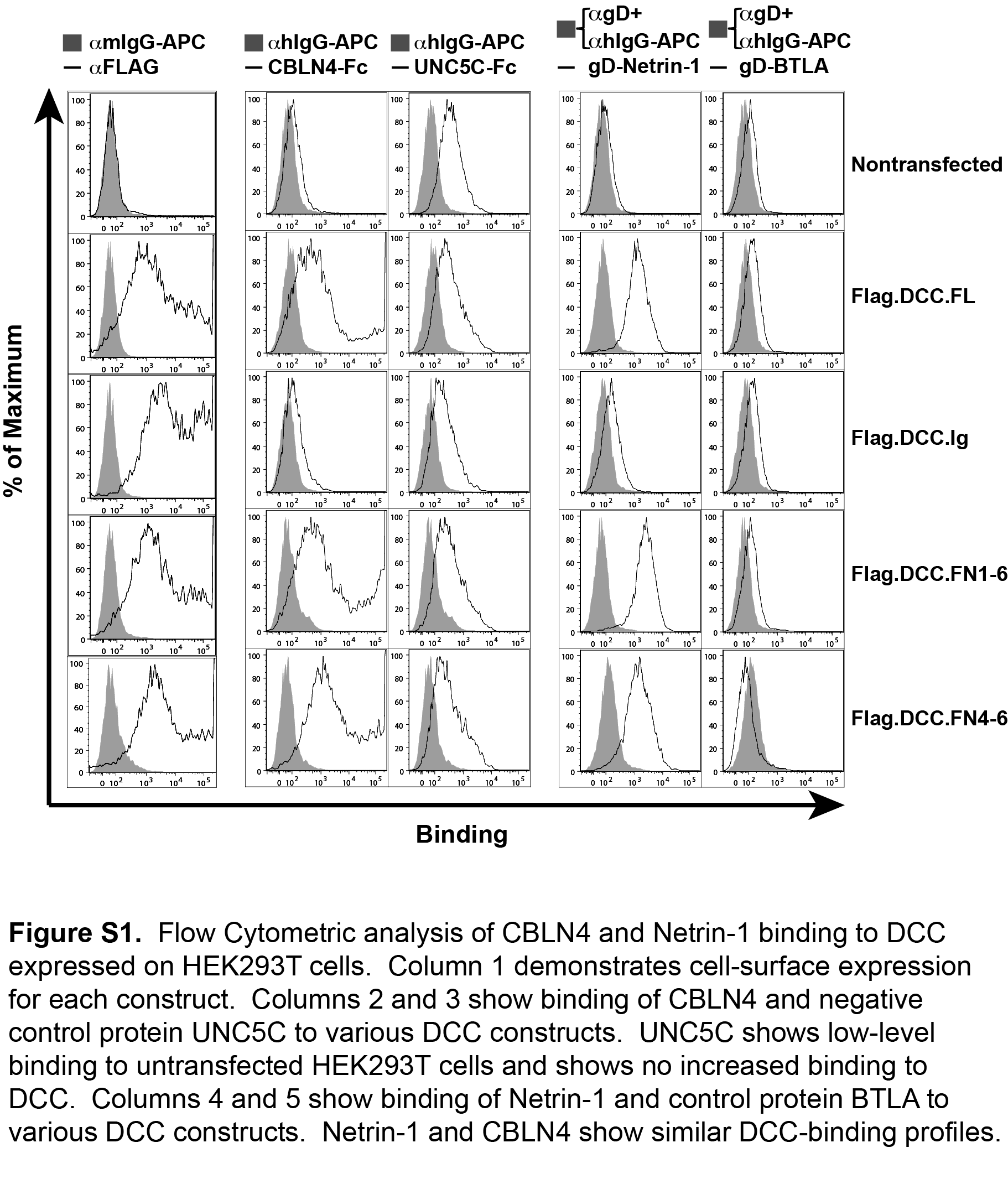

Supplement: Figure S1 — Flow Cytometric analysis of CBLN4 and Netrin-1 binding to DCC expressed on HEK293T cells. Column 1 demonstrates cell-surface expression for each construct. Columns 2 and 3 show binding of CBLN4 and negative control protein UNC5C to various DCC constructs. UNC5C shows low-level binding to untransfected HEK293T cells and shows no increased binding to DCC. Columns 4 and 5 show binding of Netrin-1 and control protein BTLA to various DCC constructs. Netrin-1 and CBLN4 show similar DCC-binding profiles. (TIF) [file pone.0084823.s001.tif]
